# Insights into morphological and physio-biochemical adaptive responses in mungbean (*Vigna radiata* L.) under heat stress

**DOI:** 10.3389/fgene.2023.1206451

**Published:** 2023-06-15

**Authors:** Ragini Bhardwaj, Jafar K. Lone, Renu Pandey, Nupur Mondal, R. Dhandapani, Surendra Kumar Meena, Suphiya Khan, Gayacharan

**Affiliations:** ^1^ ICAR-National Bureau of Plant Genetic Resources, New Delhi, India; ^2^ Department of Bioscience and Biotechnology, Banasthali Vidyapith University, Tonk Rajasthan, India; ^3^ Division of Plant Physiology, ICAR-Indian Agricultural Research Institute, New Delhi, India; ^4^ Shivaji College, University of Delhi, New Delhi, India; ^5^ Division of Crop Improvement, ICAR-Indian Grassland and Research Institute, Jhansi, India

**Keywords:** greengram, heat stress signaling pathways, oxidative stress, mungbean germplasm, adaptative traits

## Abstract

Mungbean (*Vigna radiata* L. Wilczek) is an important food legume crop which contributes significantly to nutritional and food security of South and Southeast Asia. The crop thrives in hot and humid weather conditions, with an optimal temperature range of 28°–35°C, and is mainly cultivated under rainfed environments. However, the rising global temperature has posed a serious threat to mungbean cultivation. Optimal temperature is a vital factor in cellular processes, and every crop species has evolved with its specific temperature tolerance ability. Moreover, variation within a crop species is inevitable, given the diverse environmental conditions under which it has evolved. For instance, various mungbean germplasm can grow and produce seeds in extreme ambient temperatures as low as 20°C or as high as 45°C. This range of variation in mungbean germplasm for heat tolerance plays a crucial role in developing heat tolerant and high yielding mungbean cultivars. However, heat tolerance is a complex mechanism which is extensively discussed in this manuscript; and at the same time individual genotypes have evolved with various ways of heat stress tolerance. Therefore, to enhance understanding towards such variability in mungbean germplasm, we studied morphological, anatomical, physiological, and biochemical traits which are responsive to heat stress in plants with more relevance to mungbean. Understanding heat stress tolerance attributing traits will help in identification of corresponding regulatory networks and associated genes, which will further help in devising suitable strategies to enhance heat tolerance in mungbean. The major pathways responsible for heat stress tolerance in plants are also discussed.

## 1 Introduction

Mungbean (*Vigna radiata* L. Wilczek), also commonly called as greengram, is a leguminous crop. It is an annual grain legume crop, cultivated in different soil types of the South-East Asia and South East Africa, Australia, and South America (Parihar et al., 2017). The crop requires warm-humid climatic conditions, with temperature ranging between 25°C and 35°C and a well distributed rainfall of 400–550 mm during growing season. Mungbean has a high range of storage protein (22%–27%) with sugar, minerals, and soluble dietary fibers (Alom et al., 2014). Recently, the crop area and production has increased demand of plant-based protein with affordable market price, as a result, mungbean is now being commercially cultivated in large scale (Keatinge et al., 2011). Global production of mungbean is around 6.0 million tones which comes from a cultivated area of about 7.3 million hectares (Gayacharan et al., 2023). India alone produces mungbean up to 41% of the global production which makes it the largest producer of mungbean followed by Myanmar, Bangladesh and Pakistan (Schreinemachers et al., 2019). Loam to sandy loam soils with good drainage are the best suited for mungbean cultivation. Because of its short life span, nitrogen-fixing ability, low water requirement, great biomass, and high yield mungbean is considered as one of the most important crops in agriculture (Alom et al., 2014). However, the high variability in climatic conditions including rising temperature and unpredictable water deficit environments during its cropping season cause drastic reduction in mungbean productivity (Singh et al., 2016). Several abiotic stresses such as heat, salinity, water-logging, and drought highly affects the growth and development in Mungbean (Dreesen et al., 2012; Bita and Gerats, 2013; Kaur and Nayyar, 2015; Landi et al., 2017; Zandalinas et al., 2017).

Among various factors, global temperature rise is the major challenge in legume crop production. The erratic and low rainfall, soil desertification, evolving new races of pest and pathogens are some other problems associated with the global temperature rise, which are adversely impacting crop production across the globe. Amid climate change, cultivation of legume crops has become more challenging, as these are obligatory adapted to low input environments and are majorly cultivated in rainfed conditions. Legumes are highly impacted by insect, pest primarily attributed to their protein rich nature and narrow genetic base. The regional report of Middle East and North Africa (MENA) region, climate change adversely impacts pulses more than any other crop group (Njuki et al., 2022). The regional report on MENA region indicated yield reduction due to climate change in pulses by 17.2%, followed by oil seeds (6.86%), cereals (4.18%) and fruits and vegetables (1.78%) (Njuki et al., 2022). Similar impact of climate change is observed across the globe with varying severity. However, among all food sources, pulses are the only food for which increased consumption demand is predicted by 2050 (Njuki et al., 2022).

Legumes including mungbean, which are grown in warm-humid climatic conditions are more affected by high temperature. In India, mungbean cultivation during summer season faces severe heat waves, and sometime temperature rises to 45°C, at which most of the cellular processes stop functioning. Various studies have demonstrated that under heat stress, significant yield losses occur in mungbean at reproductive stage of plant (Hamada, 2001; Hall, 2010; Kumar et al., 2013; Kaur and Nayyar, 2015; Sharma et al., 2016; Priya et al., 2020). Studies also described that mungbean under heat stress at reproductive stage is more adversely impacted as compared to vegetative stage under heat stress (Hall, 2010; Priya et al., 2020). Moreover, the male reproductive parts are more at risk to heat stress in comparison to the female reproductive parts in mungbean (Dickson and Boettger, 1984; Monterroso and Wien, 1990). Further, it is also found that physiological processes in reproductive tissues of mungbean are more susceptible to heat stress (Asseng et al., 2011; Kaur et al., 2015).

Heat stress triggers numerous physiological and biochemical processes in mungbean to counter the heat stress impact, but the crop yield is drastically reduced when severity of the heat stress is extreme (HanumanthaRao et al., 2016; Sita et al., 2017). However, very less research has been done to understand the impact of heat stress on mungbean for yield attributing traits and the reproductive parts. Therefore, to increase the productivity of mungbean under heat stress environment, it is important to find out the physio-biochemical and molecular variations for high temperature stress tolerance in the mungbean germplasm, and probe the mechanisms leading to heat sensitivity in mungbean crop. In this review, we provide recent understanding of heat stress effects and tolerance mechanisms in mungbean, focusing on its morphological, and physio-biochemical responses under high temperature stress.

## 2 Impacts of heat stress in mungbean

**FIGURE 1:**
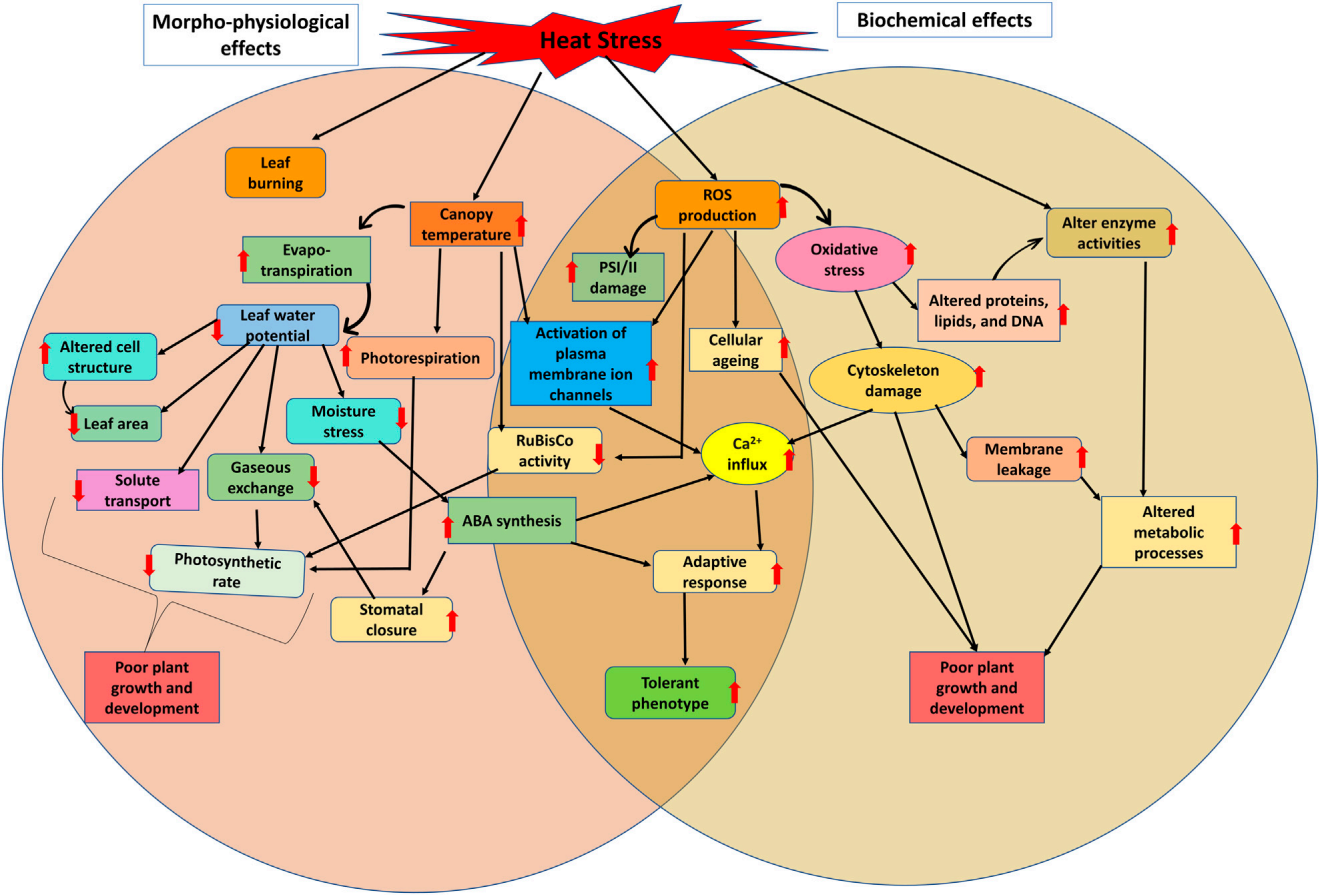
General overview of impact of heat stress on physiological and metabolic pathways including morphological changes in plants under heat stress condition.

**FIGURE 2:**
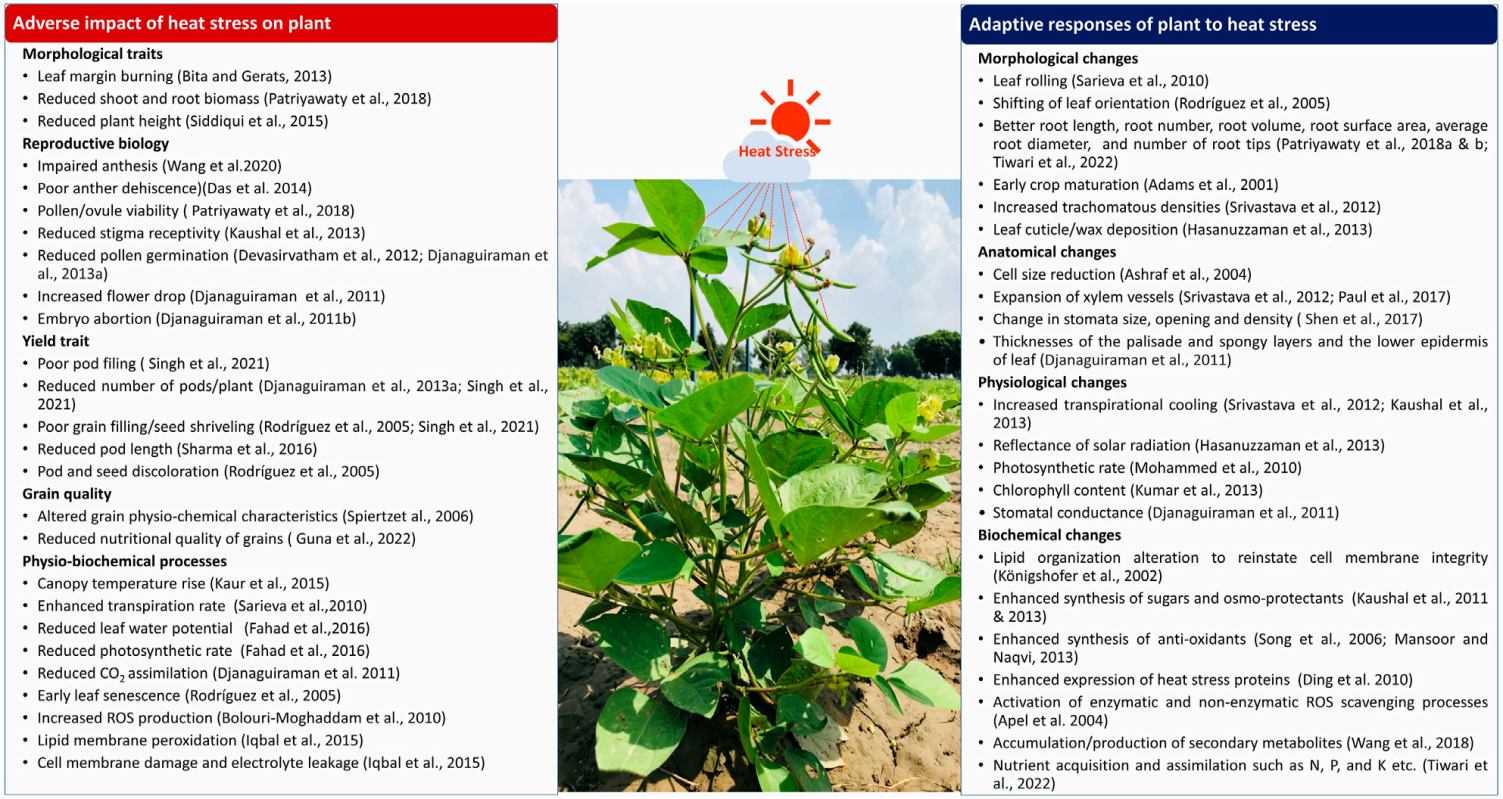
Impact of heat stress on morpho-anatomical features, reproductive biology and physio-biochemical properties of plants under heat stress. The corresponding adaptive responses of the plant under stress are also highlighted.

Mungbean is a summer season crop that may be cultivated in all dry and semi-arid parts of the world. However, recent global average temperature rise has posed a threat for the mungbean crop production (Abd El Lateff et al., 2018). The constant higher atmospheric temperature for longer duration is highly detrimental for the growth and physiological functions of various food crops (Cao et al., 2011). Severity of the crop damage varies with the timing, duration and magnitude of the elevated temperature, as well as the genotype specific defense response. In mungbean, during the summer season where temperature rise above 40°C causes terminal heat stress during reproductive stage of the plants which is a major concern in mungbean productivity because it results in impaired anthesis, loss of pollen viability, reduced flower fertilization, increased flower drop and shortened period for grain filling (HanumanthaRao et al., 2016; Basu et al., 2019) (Figure 1; Figure 2). Even an increase in temperature by a few degrees changes crop cycle and accelerates flower drop and embryo abortion, and poor grain filling (Kaur and Nayyar, 2015; Kaushal et al., 2016). Also, in kharif season mungbean temperature >40°C occur during early growth stages of the crop, causing similar problem particularly in northern parts of India (HanumanthaRao et al., 2016).

Reproductive organs are highly sensitive to high temperature stress. In mungbean, high temperature cause flower shedding as high as 79% (Kumari and Varma, 1983). However, the genotypic variability in mungbean germplasm is observed attributing to specific or combination of heat stress tolerance mechanisms (Kaur et al., 2015; Baroowa et al., 2016; Sharma et al., 2016). The effect of heat stress in mungbean is not thoroughly investigated yet, and it needs more in-depth research (Kaur et al., 2015).

### 2.1 Morphological and anatomical changes in mungbean in response to heat stress

**FIGURE 3:**
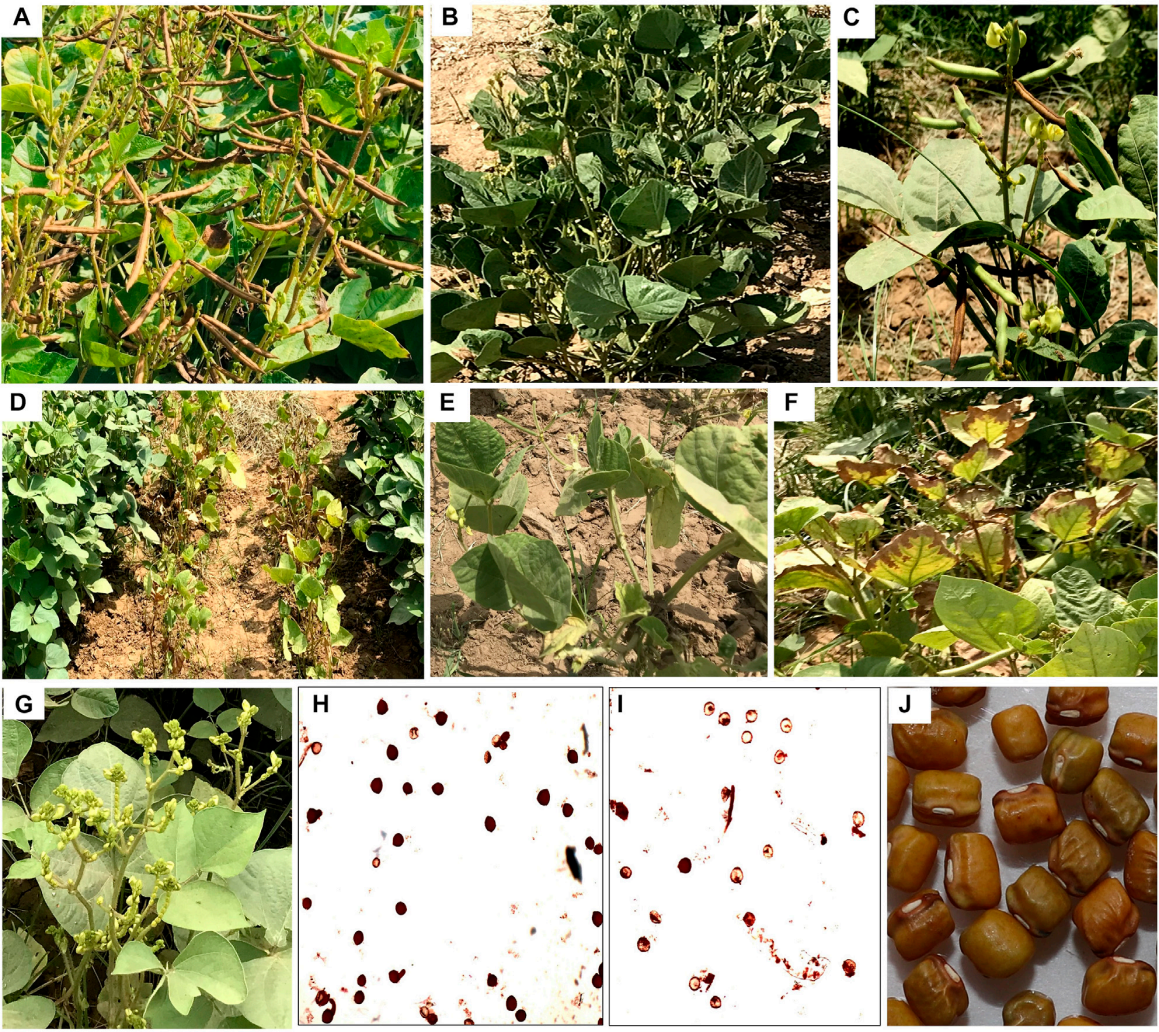
Typical morphological symptoms in mungbean plants in response to heat stress. Each genotype has a specific response to heat stress, e.g., flower drop, poor grain filling, pod discoloration, and leaf margin burning **(A)**, upward curvature of leaves to protect plant from heat from sunlight **(B)**, poor pod filling and reduced pod length **(C)**, early onset of maturity **(D)**, leaf abscission due to early onset of senescence to mobilize nutrients towards reproductive parts **(E)**, leaf margin burning **(F)**, almost entire flower drops due to high temperature during reproductive phase **(G)**, heat tolerant genotype with better pollen viability **(H)** than the heat susceptible one **(I)**, and seed shriveling due to heat stress during grain filling stage **(J)**.

Heat stress can cause a range of modifications at morphological and anatomical levels in plants such as scorching of leaves and stems, loss of leaves, inhibition in shoots and roots growth, shrinking of seeds, and damage to fruits. As a result of these alterations, it consequently lead to reduced crop productivity (Vollenweider and Günthardt-Goerg, 2005). In mungbean, heat stress causes many structural changes at morpho-anatomical level (Figures 3A–H). The different phases of reproductive stage such as pollen germination, loss of pollen viability, less pollen load on stigma, poor anther dehiscence, pollen sterility, and poor ovule viability lowers the crop yield (Kaushal et al., 2013). In a study by Kaur et al. (2015) on two mungbean genotypes (SML 832 and SML 668), similar results were observed in response to heat stress treatment. They also observed decrease in plant biomass (16%–19%), total number of pod set, seed yield (35%–40%), number of filled pods (32%–38%), and seed number (43%–47%), due to high temperature exposure.

Failure of ovule fertilization is often associated with plant productivity factors such as loss of pollen viability, loss of stigma receptivity and reduced pollen tube growth (Hurkman et al., 2009; Patriyawaty et al., 2018). Pollen becomes often non-viable before fertilizing the flower (Figures 3I,J). It is also reported that the higher ambient temperature reduces the stigma receptivity to pollen causing embryo abortion and poor seed set (Kaushal et al., 2013). Reductions in seed set per pod are reported to be correlated with the poor pollen tube growth and tropism defects (Zinn et al., 2010). Moreover, heat stress can directly damage cell membranes, leading to changes in their permeability. The heat stress can alter the microtubules organization which not only affect the elongation, differentiation, and expansion of cells but also negatively impacts the cytoskeleton structure (Rasheed, 2009; Bita and Gerats, 2013). In addition, heat stress also causes decrease in pod length, seed quality, seed size, and number of seeds per pod which ultimately reduces grain yield and quality in mungbean. Similarly, Sharma et al. (2016) reported that heat stress-induced oxidative stress caused chlorosis, leaf rolling, and leaf blistering in mungbean plants.

Several studies have confirmed the susceptibility of mungbean to rising temperatures (Jha et al., 2017). High-temperature stress can have drastic impacts on plant growth and development, as well as on different physiological activities (HanumanthaRao et al., 2016). For instance, mungbean may lose vigor due to extended exposure to high temperatures results in limiting seedling growth and development (Kumar et al., 2011; Devasirvatham et al., 2012). Heat stress can also result in negative impacts on vegetative growth, including leaf senescence, chlorosis, necrosis, burning, and abscission, reduced internode elongation, and suppression of root and shoot development (Kaushal et al., 2016; Sharma et al., 2016). Other effects of heat stress on mungbean include leaf curling, plant wilting, yellowing and blackening of leaves, reduction in plant height, reduced number of branches, and biomass (Kaur et al., 2015). Figure 2 and Table 1 illustrate the impact of heat stress on mungbean’s morphological and anatomical traits.

**TABLE 1:** Sources of heat stress tolerance identified in mungbean for various agro-morphological, physiological and biochemical traits.

Heat tolerant genotypes identified	Heat stress tolerance attributing traits	References
Morphological traits		
EC693369, EC693358, EC693357, ML1299 and Harsha	Reduction in leaf area	Sharma et al. (2016)
EC398889	Pollen fertility, sucrose-synthase activity and photosynthesis at elevated temperature	Basu et al. (2019)
ML-2037	Number of pods/plant, number of seeds/pod, and seed weight	Singh et al. (2021)
AUSTRC 324277, Celera, Jade-Au, Satin II, and White Gold	Physiological parameters, grain yield, and shoot biomass	Patriyawaty et al. (2018)
SML832	Plant biomass	Kaur et al. (2015)
MH-421, MH-318, and Basanti	Higher yield	Chand et al. (2020)
**Physiological traits**		
Samrat, IPM 02-3	Better chlorophyll stability index, higher stomatal conductance	Solai et al. (2015)
SML832	Increased canopy temperature depression	Kaur et al. (2015)
VGG 17004, VGG 17003, VGG 15029, VGG 16069, and COGG 1332	Relative water content, chlorophyll stability index, and total chlorophyll content	Jincy et al. (2019)
VGG 17019, VGG 17010, ARM 1, VGG 17004, VGG 17006, VGG 17003, and VGG 15029	Total chlorophyll content, RWC and chlorophyll stability index	Jincy et al. (2020)
SML832	Chlorophyll content, canopy temperature depression, Photosynthetic rate	Kaur et al. (2015)
Ganga 8, IPM 06-5, IPM 03-3, IPM 409-4, IPM-02-3, MH-736, MH-805, MH-421, MH-810, and MH-721	Higher CTD, MSI, and total chlorophyll content	Punia et al. (2019)
**Biochemical traits**		
Harsha, EC693369, EC693357, ML1299, and EC693358	Increased superoxide dismutase, glutathione reductase activity, ascorbate peroxidase and catalase activity	Sharma et al. (2016)
EC 398889	Sucrose-synthase activity at elevated temperature	Basu et al. (2019)
SML668	Decreased malondialdehyde and H_2_O_2_ contents	Kumar et al. (2011)
NCM-89, NM-20-21, NM-19-19, and NM-121-123	Decreased lipid peroxidation, increased peroxidase activity	Mansoor and Naqvi (2013)
MH-421, MH-318, and Basanti	Increased proline and soluble carbohydrate contents	Chand et al. (2020)

### 2.2 Physiological changes in mungbean under heat stress

Heat stress can have several negative impacts on plant physiology, such as chlorophyll reduction, decreased photosynthesis, decreased transpiration, increased canopy temperature, and increased stomatal aperture (Schoffl et al., 1998; Kaushal et al., 2013). These effects can ultimately lead to reduced plant productivity. Additionally, heat stress can cause membrane damage, protein degradation, and altered metabolism in plant cells (Schoffl et al., 1998; Kaushal et al., 2013). Heat stress can also increase electrolyte leakage in plants due to altered membrane permeability by direct injuries, which affects the differentiation, elongation, and expansion of cells (Rasheed, 2009; Bita and Gerats, 2013). Further, structural alterations in chloroplast protein complexes and reduction in enzyme activity occur due to the initial impacts of thermal stress (Ahmad et al., 2010). Although a modest rise in ambient temperature generally promotes plant growth and development, it shortens the plant’s life span and results in a significant reduction in light uptake during the plant’s growth phase (Kalaji et al., 2016). Additionally, plant water status is a critical component of plant survival under heat stress. Plants try to normalize their canopy temperature through increased transpiration rate, and therefore, heat stress in combination with soil moisture stress proves to be most detrimental to the plant (Simões-Araújo et al., 2003). Unfortunately, most of the legume crops, including mungbean, are cultivated under rainfed conditions in tropical and sub-tropical regions. As a result, increasing global warming and adverse climatic conditions disrupt the monsoon seasons, resulting in uneven/less rainfall, which affects plant growth and development (Simões-Araújo et al., 2003). Heat stress can rapidly reduce tissue water content despite ample availability of soil moisture, similar to drought stress conditions, leading to a disruption in nutrient uptake from roots (Wahid et al., 2007). The drastic water loss due to high transpiration, particularly during the daytime, affects important physiological processes, ultimately resulting in reduced plant growth and development (Fahad et al., 2017). Heat stress can also reduce seed viability and lower plant and yield quality. Moreover, plants exhibit programmed cell death in some specific cells and tissues under heat stress (Anderson and Padhye, 2004).

In mungbean, the ideal temperature for growth and development is 28°C–30°C, and further every degree increase in temperature may reduce crop production by 35%–40% (Tzudir et al., 2014; HanumanthaRao et al., 2016; Sharma et al., 2016). Mungbean plant can thrive well up to 40°C of temperature, after which flower shedding begins (Zinn et al., 2010; Sita et al., 2017). Heat stress reduces leaf area and stomata openings which cause dramatic reduction in the carbon dioxide assimilation rate and photosynthesis during the vegetative stage of the mungbean (HanumanthaRao et al., 2016). The increase in carbon dioxide (CO_2_) content also causes stomatal closure, which hinders photosynthesis in the mungbean. High CO_2_ concentration along with high ambient temperature, proves more detrimental for mungbean growth and development (Reardon and Qaderi, 2017). In a study, CO_2_ assimilation in mungbean was significantly reduced at 40°C, which had a direct impact on photosynthetic efficiency (Karim et al., 2003). High temperatures also reported to reduce the chlorophyll and carotenoid levels, as well as the chlorophyll stability index (Chand et al., 2018). A slightly higher temperature (36°C) temperature treatment on mungbean genotypes indicated that the exposure of plants to high temperature has greater adverse impact on leaf conductance at the pre-flowering stage than the blooming and grain filling stages, although high-temperature treatments had no effect on transpiration rate at any stage, but photosynthetic activity decreased at all three stages (Islam, 2015). Similarly, another study on three prominent mungbean varieties viz., MH 421, MH 318, and Basanti indicated reduction in chlorophyll and carotenoid content, as well as a decrease in chlorophyll stability index in response to high temperature (Chand et al., 2018). Under heat stress, sensitive genotypes (MH 318 and Basanti) showed higher losses in the physiological attributes stated above, whereas tolerant genotypes (MH 421) maintained high yield and physiological functioning (Chand et al., 2018). Evidenced from numerous studies it is found that Photosynthesis is highly sensitive to high temperatures (Sinsawat et al., 2004). Heat stress affects photosynthetic functions in mungbean by disrupting photosynthetic machinery, causing structural aberrations (particularly the thylakoid membrane) and alterations of chloroplast enzymes. Based on mungbean germplasm screening for heat responsive traits various important promising mungbean donors are identified (Table 1). Also, impact of heat stress on physiological processes in various crops is listed in Table 1 and Figure 1, Figure 2.

### 2.3 Biochemical changes in mungbean under heat stress

High temperatures have a significant impact on the metabolism and biochemistry of mungbean plants. Under heat stress, the formation of reactive oxygen species (ROS), such as hydroxyl radical, singlet oxygen, superoxide radical and hydrogen peroxide, increases, causing protein degradation, membrane damage, and enzyme inactivation, and hence increases oxidative stress (Liu and Huang, 2000). Long-term exposure to relatively high temperature stress can result in severe cellular injury or death, while at extremely high temperatures, this can occur in just minutes (Wahid et al., 2007). These injuries, coupled with a lack of water content, can cause a reduction in ion flow and plant growth, as well as an increase in the generation of toxic compounds and reactive oxygen species (Howarth, 2005). Some of the metabolic effects of heat stress on mungbean plants are briefly described in Figure 1 and Table 2.

**TABLE 2:** Candidate genes identified under heat stress tolerance in plants for various agro-morphological, physiological and biochemical traits

Traits	QTLs/Genes involved	Regulation in response to heat stress	References
Morphological/anatomical traits			
Pollen viability	*PolVia5.1, PolVia8.1*	15%–16% and 20% of phenotypic variation respectively	Vargas et al. (2021)
Pollen development	*Hsp100, Hsp90, Hsp70, Hsp60, smHsp*	Reduce heat stress impact on pollen grain development by protecting cellular proteins from denaturation to retain their function	Kotak et al. (2007)
	*HSFA2a, HSFA2d, HSFA2f, HSFA9, HSFA3, HSFB2a, HSFB2b, HSFB2c*	Helps in pollen development in plants under heat stress	Zhang et al. (2012), Giorno et al. (2013)
Pollen germination	*Ca_24217*	Reduced activity	Jha et al. (2021)
Pollen tube elongation	*Ca_14063*	Reduced activity	
Root length	*Ca_07091*	Increased activity	
Stigma receptivity	*FER/AAK1/SIR/SRN*	Decreased activity	Escobar-Restrepo et al. (2007)
Flower abscission	*RAP2-11/ERF002*	Increased activity	Chen and Chen (2002)
Number of filled pods	*qfpod02_5*	Cumulative phenotypic variation explained above 50%	Paul et al. (2018)
Grain yield per plot	*qgy02_5*		
Total number of seeds per plot	*qts02_5*		
% pod setting	*q%podset06_5*		
Grain filling	*PdShr1.1*	Increased activity	Vargas et al. (2021)
	*SuSy (sucrose-synthase)*	Poor pod filling and pollen development and germination	Basu et al. (2019)
	*ERF3, GBSS1, GW2, ERL1*	Panicle compactness, and ethylene production negatively influences the grain filling	Sekhar et al. (2021)
Discoloration of seed coat colour	*Hbs-1, Hbs-2, Hbs-3*	Ethylene forming enzymes including ACC oxidase 2 and ACC synthase 1	Pottorff et al. (2014)
Nodule thermotolerance	*VuNSR10*	Negative regulation of Pherophorin-like protein which plays a role in cell wall structure	Simões-Araújo et al. (2002)
	*VuNSR11*	Positive regulation of *Xylan endohydrolase* which plays a role in cell wall structure	Simões-Araújo et al. (2002)
**Physiological traits**			
Transpiration rate	*Qe.ccshau-4A*	Increased activity	Sangwan et al. (2019)
Membrane stability index	*CaCMS_NS4.1*	Increased activity	Jha et al. (2021)
Chlorophyll content	*QLCCHR.nri-4A*	Increased activity	Maulana et al. (2018)
	*CaCHL_NS4.3*	Reduced activity	Jha et al. (2021)
Stomatal conductance	*RAN1/HMA7*	Reduced activity	Chen and Chen (2002)
Stomatal development	*HSP90s*	HSP90s interact with YODA cascade, affects phosphorylation of SPEECHLESS (SPCH) and MPK6 to modulate stomatal development	Samakovli et al. (2020)
Canopy temperature depression (CTD)	*QCtdh.tam-3B*	Comparatively higher activity in heat tolerant genotypes	Mason et al. (2013)
Photosynthetic rate	*Qpn.ccshau-2.1D*	Comparatively higher activity in heat tolerant genotypes	Sangwan et al. (2019)
Leaf senescence	*Ca_12767*	Comparatively lower activity in heat tolerant genotypes	Jha et al. (2021)
**Biochemical traits**			
Late embryogenesis abundant (LEA) proteins	*VrLEA-2, VrLEA-40, VrLEA-47, and VrLEA-55*	Higher expression in heat tolerant genotypes	Singh et al. (2022)
hydrogen peroxide	*Ca_17121*	Reduced activity	Jha et al. (2021)
Superoxide dismutase	*Cu/Zn SOD*	Increased activity	Paul et al. (2018)
Catalase	*CAT1*	Increased activity	Ara et al. (2013)
Gluathathione reductase	*Zm00001d027769*	Increased activity	Sheoran et al. (2022)
Ascorbate peroxidase (APX)	*CaAPX*	Increased activity	Wang et al. (2017)
Peroxidase	*Zm00001d028347*	Increased activity	Sheoran et al. (2022)

In mungbean, high temperature especially >40°/30°C (max/min) reduces leaf water potential and increases oxidative stress resulting in growth suppression and chlorosis which is linked to reduced crop yield (Kumar and Wigge, 2010). High temperature stress can cause a considerable increase in hydrogen peroxide (H_2_O_2_) concentration in mungbean as well as in other plants, which could be due to a reduction in catalase activity by blocking catalase production which lowers the enzyme’s steady-state level because of high turnover rate (Scandalios et al., 1997). When plants are exposed to high levels of heat, they experience an oxidative burst (N, 1997) that can cause an increase in H_2_O_2_ (Levine et al., 1994; Baker and Orlandi, 1995). Antioxidant enzymes such as SOD and CAT become less active during heat shock, compromising the plant’s defenses and leading to increased levels of oxidant species (Willekens et al., 1995; Foyer et al., 1997; Polle, 1997). This can have a negative impact on cellular metabolism, resulting in high levels of harmful compounds like malondialdehyde and H_2_O_2_ that affect plant productivity (Bita and Gerats, 2013). Lipid peroxidation under aerobic conditions, is a natural metabolic process that can be affected by ROS, causing damage to the cell membrane and impairing its function (Blokhina et al., 2003). (Heath and Packer, 1968). The presence of malondialdehyde (MDA) is an indication of oxidative damage, and it is created by the lipid peroxidation of the cell membrane (Mandhania et al., 2006). It has been reported that high temperature stress on four mungbean genotypes (NCM 89, NM 20-21, NM 121-123, and NM 19-19) at seedling stage observed an increase in the levels of lipid peroxidation as there is a considerable increase in MDA concentration (Mansoor and Naqvi, 2013). Studies in seedlings of four mungbean genotypes have shown that an increase in temperature can cause an increase in lipid peroxidation, leading to decreased net photosynthesis, water use efficiency, stomatal conductance, total chlorophyll, and nutrient partitioning in sensitive genotypes. Additionally, heat stress can negatively impact assimilate partitioning and apoplastic to symplastic phloem transport, resulting in a decrease in carbohydrate buildup and viability of pollen grains in mungbean (Kaur et al., 2015; Taiz et al., 2015; Hanif and Wahid, 2018).

Seed shriveling in legumes is primarily linked to reduced synthesis of carbohydrates and storage proteins due to elevated temperature. The four enzymes viz., Adenosine Diphosphate Glucose Pyrophosphorylase, Starch Branching Enzyme, Starch Synthase, and Sucrose Synthase are crucial for the grain filling process (Taiz et al., 2015). In general Sucrose synthase plays in important role in grain filling (Basu et al., 2019), and temperature beyond a certain period affects the enzyme activities and lead to poor grain filling. Similarly, the functioning of Nitrate reductase is also diminished during heat stress, which is a most important enzyme of nitrogen (N) metabolism in plants. It catalyzes the reduction of inorganic N in the form of nitrate to organic form in translational process of proteins. Heat stress is known to adversely affect the nitrate reductase enzyme activity, which is more detrimental for leguminous crops affecting protein biosynthesis during grain filling and reproductive stages (Klimenko et al., 2006; Farooq et al., 2017). Heat stress also impairs the nitrogen-fixing activity of mungbean by restricting the production of root hair and infection thread (Bansal et al., 2014). Furthermore, heat stress reduces seed viability, plant and seed quality (Anderson and Padhye, 2004). The nutritional value of seed is impaired mainly due to adverse impact of heat stress on synthesis and accumulation of protein and carbohydrates (Taiz and Zeiger, 2010).

## 3 Adaptation strategies in mungbean to develop heat tolerant genotypes

**FIGURE 4:**
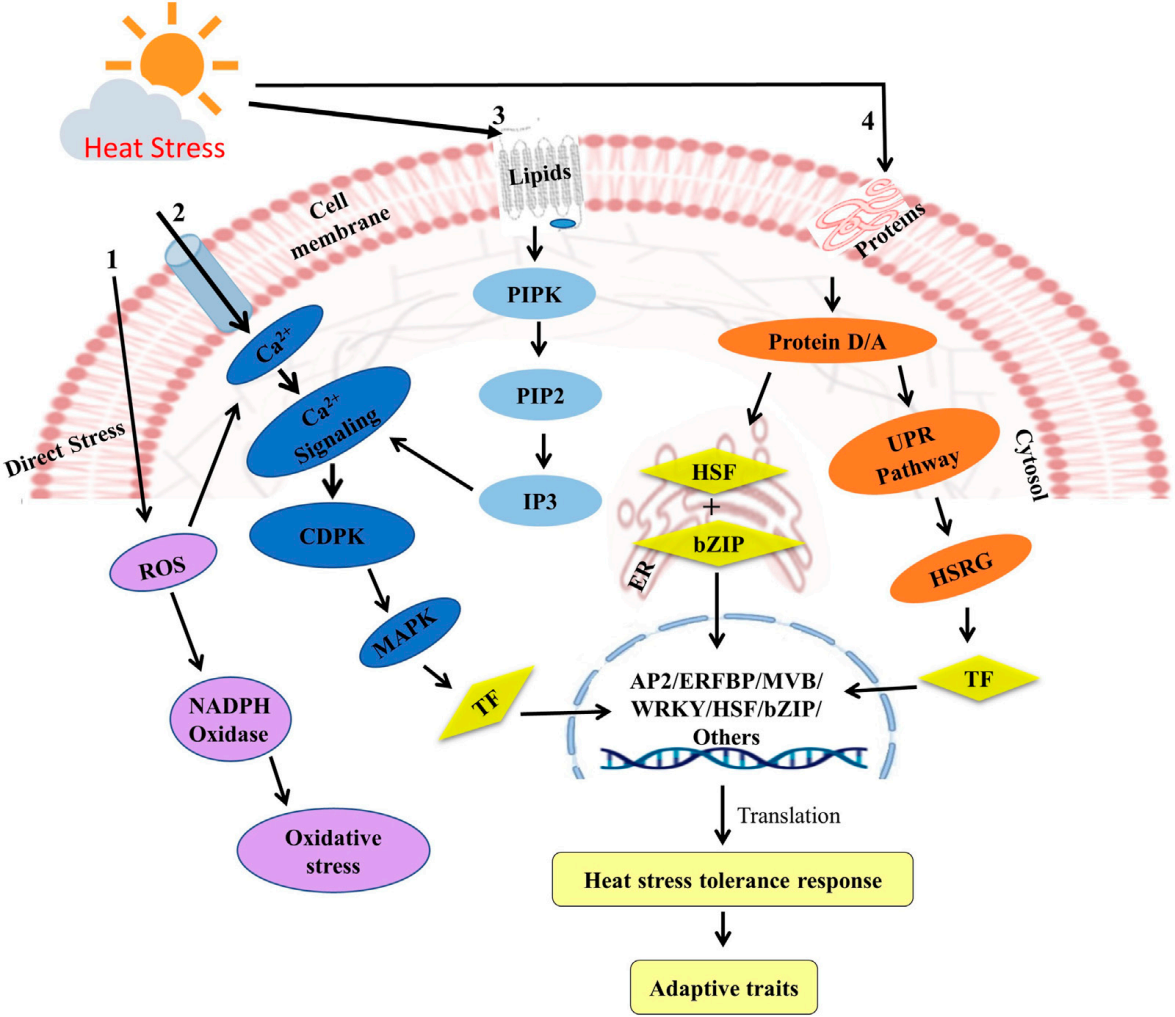
Different signaling and defense pathways in response to heat stress in plants. High temperature stress effects the plasma membrane to activate the calcium channels, which induces Ca^2+^ influx, thus the MAPK cascade regulates and activates the various transcriptional factors which ultimately leads the accumulation of the stress responsive genes, the antioxidants and ROS.

Mungbean plant deploy a number of adaptation mechanisms such as changes in plant morphological and anatomical features, secondary metabolite production and their accumulation in target tissues/cells, production of anti-oxidants, stress hormones and proteins, and alteration in myriad of physiological activities (Figure 2). The adaptation mechanisms that result in thermal tolerance in plants include secondary metabolites, heat-shock proteins (HSPs), ROS scavenging systems, and accumulation of suitable solutes (Nakamoto and Hiyama, 1999; Wahid et al., 2007; Mittler et al., 2012; Hanif and Wahid, 2018). During heat stress, ROS produced by aerobic metabolism have a deleterious impact on cellular catabolism, causing lipid membrane peroxidation as well as damage to proteins and nucleic acids (Bita and Gerats, 2013). To tolerate high temperatures, plants need to have sufficient levels of antioxidants (Awasthi et al., 2015). When subjected to heat stress, plants generate and obtain new stress proteins, such as HSPs. These HSPs are molecular chaperones that play a crucial role in protein folding, translocation, proper aggregation, and degradation under both normal and stressful conditions (Vierling, 1991). HSP100, HSP90, HSP70, HSP60, and the small heat-stress protein (sHSP) family (Wang et al., 2004) are five heat-stress protein/chaperone families that play an important role in mitigating heat-stress, including protecting native proteins against denaturation. Isoprenoids, flavonoids, and carotenoids are secondary metabolites that inhibit peroxidase activity and promote high-temperature stress tolerance (Havaux, 1998; Loreto et al., 1998; Rivero et al., 2004). When plants are exposed to heat stress, they undergo various interconnected morpho-physiological, biochemical, and molecular changes unique to their tolerance and adaptive nature to their surrounding environment, ultimately enabling them to restore redox homoeostasis (Waseem et al., 2011) (Figure 4). This indicates the necessity in developing heat stress tolerance in mungbean cultivars. Mungbean plants have adapted various types of defense mechanism to survive the heat stress, which are discussed in the following sections. Sources of tolerance identified by various workers are highlighted in Table 1.

### 3.1 Morphological and anatomical adaptations involved in mungbean response to heat stress

Increase in atmospheric temperature beyond a certain optimum point drastically reduces the plant growth and development as cellular processes require a specific temperature range. Plants have evolved with a great variability in their morphological adaptation as a part of survival mechanisms, such as early maturation, leaf rolling, and changing leaf orientation to cope up with the certain level of temperature deviations (Srivastava et al., 2012). However, these adaptation mechanisms are often associated with yield losses under high temperature in many crop plants (Adams et al., 2001; Rodriguez et al., 2008). Further rise in atmospheric temperature causes development of symptoms such as scorching of leaves and twigs, shoot and root growth inhibition, early onset of leaf senescence and abscission, and pod discoloration as an adaptation for plant survival. In response to heat stress, plant height is reduced in mungbean, which is specifically attributed to the disruption in cell elongation. This demonstrates that the mungbean plants exhibit the adaptation mechanism to respond heat stimuli. Certain important differences can be detected in the parameters like 100 seed weight, seeds/plant, pods/plant, seed yield, total biomass/plant, branches/plant, and plant height, etc. In mungbean as adaptation traits (Sharma et al., 2016) (Table 1). The reproductive phase of plant growth and development is highly sensitive to heat stress. To overcome certain level of higher temperature, plant deploy two very crucial mechanisms of plant defense viz. Dehydration of pollen grains and embryos, resulting in stress tolerance in the pollen grains, and prolonged dormancy in embryos under stressful conditions (Zinn et al., 2010). As a result, pollen and embryos remain viable under higher temperature, however, the magnitude of tolerance varies among genotypes. Therefore, these traits are considered as important for development of heat-tolerant mungbean varieties (Rainey and Griffiths, 2005).

In a study effects of heat stress on 41 mungbean genotypes for their vegetative and reproductive activities under controlled growing conditions was examined and a few tolerant lines for stress were identified (Sharma et al., 2016). A field study in New Delhi in the Kharif seasons of 2014 and 2015 was conducted to assess the impact of high temperatures on seven mungbean genotypes in a rain-fed environment, and various heat stress responsive morphological traits were identified (Chikukura et al., 2017). Night temperature rise is considered as more crucial for flower drop, as it is observed that under the higher temperature stress, the flower abscission is higher during the night as compared to the daytime (Solai et al., 2015). Under high temperature stress, plants maintain their metabolites level required for flower and pollen and anther development. According to Banon et al. (2004), the reduction of cell size and the closure of stomata can help to decrease excessive water loss and lead to increased stomatal density and enlarged xylem vessels in plants under heat stress. However, Shen et al. (2017) have noted that the anatomical changes in response to heat stress can vary among different species. Some of the important morphological, physiological, biochemical and anatomical responses of mungbean genotypes to heat stress tolerance are highlighted in Figure 1, Figure 2 and Table 2.

### 3.2 Physiological adaptation in response to heat stress

There are few studies on how high temperature stress affects stage-specific functional physiology in legumes from post-flowering to blooming. The reproductive stage is destined to be more impacted by and prone to temperature vagaries due to its delicate organelle constitution, even if heat stress sensitivity in plants changes with plant growth. Depending on the species and genotype, there are significant inter- and intra-specific variations that affect the response under heat stress (Sakata and Higashitani, 2008; Bita and Gerats, 2013).

The persistence of photosynthesis in plants when they are exposed to stress conditions is supported by the stay-green (SGR) trait, which is also known as delayed leaf senescence. The maintenance of photosynthetic activity in plants under stressful conditions is facilitated by the stay-green (SGR) trait, also referred to as delayed leaf senescence (de Souza Luche et al., 2015). Understanding the role of SGR in plants could lead to improved plant production and productivity (Thomas and Ougham, 2014). During grain filling, the SGR characteristic allows plants to continue photosynthesizing by creating a senescence pattern that increases the amount of sugar produced by photosynthesis (Pinto et al., 2016). According to recent research, SGR wheat genotypes demonstrated improved tolerance to high temperatures due to enhanced structural stability of the photosynthetic apparatus and lower accumulation of harmful reactive oxygen species (Tian et al., 2012). The cultivar “Mairaj-2008” has also demonstrated superior ability to grow under heat stress compared to cultivars that lack the SGR trait and are more susceptible to heat damage (Nawaz et al., 2013). Canopy temperature depression (CTD) could serve as a useful tool for selecting heat-tolerant genotypes based on observable differences in their traits (Mason and Singh, 2014). Studies have shown that lower canopy temperatures promote better yield potential in wheat exposed to elevated temperatures, and CTD is effective in mitigating heat stress in wheat (Amani et al., 1996; Fischer et al., 1998; Mason et al., 2013). Examination of stomatal behavior under stress conditions can be performed using a leaf porometer, which measures fluctuations in gaseous exchange rate that trigger stomatal opening (Chandra et al., 2017). High-yielding cultivars with fully opened stomata have increased transpiration rates, improved CO2 and water vapor diffusion, and enhanced photosynthetic efficiency (Condon et al., 2007).

Chlorophyll fluorescence (ChlF) is a non-invasive marker for photosystem II (PSII) quantum efficiency and can be used to assess early stress in plants, making it a useful tool for investigating plant heat stress tolerance (Sharma et al., 2014; Kalaji et al., 2016). Studies have found that genotypes with high ChlF values, such as the heat-tolerant line RRR46 of common bean, outperform other lines when exposed to heat stress, indicating their potential for future breeding programs (Stefanov et al., 2011). Membrane stability is another important physiological trait that can affect heat tolerance in plants, and higher membrane stability during grain filling has been shown to increase heat tolerance in wheat (Gupta et al., 2013; Ramani et al., 2017).

Flag leaf photosynthetic efficiency has also been found to play a role in heat stress tolerance in crops. For example, the wheat cultivar “Jimai22”showed increased yield under heat stress and demonstrated PSII stability and significant carboxylation activity (Feng et al., 2014). Heat-tolerant mungbean and lentil genotypes have also been found to exhibit better photosynthetic efficiencies under heat stress than heat-sensitive genotypes (Sharma et al., 2016; IG3263, respectively). Conversely, decreased cellular thermostability in rice has been linked to potential reductions in crop output (Maavimani and Saraswathi, 2014). Overall, physiological traits such as ChlF, membrane stability, and photosynthetic efficiency can provide important insights into plant heat stress tolerance and can be used to identify promising genotypes for breeding programs. A comprehensive list of mungbean genotypes based on physiological traits under heat stress tolerance is provided in Table 1.

The traditional theme of research on legume reproduction has tremendously focused on vegetative stage of the plants rather than their physiological and molecular mechanisms underlying legume crop reproductive heat tolerance (Lin et al., 1984; Valliyodan and Nguyen, 2006; Kumar, 2012; Sita et al., 2017). However, recent studies have shown a growing interest in understanding these mechanisms (Devasirvatham et al., 2012; Kaushal et al., 2013; Sita et al., 2017; Patriyawaty et al., 2018). Despite this, there is still a lack of knowledge regarding the specific physiological and molecular mechanisms that allow legume crops to be resilient to heat stress during the reproductive stage (Devasirvatham et al., 2012; Kaushal et al., 2013; Sita et al., 2017; Patriyawaty et al., 2018). One possible explanation for this lack of understanding is that many legume cultivars are difficult to transform genetically, which limits the availability of transgenic evidence (Young and Udvardi, 2009; Song et al., 2013). However, on the basis of current research on other crop groups, particularly cereals, it is possible to suggest physiological and molecular explanations for legumes’ ability to withstand heat stress (Devasirvatham et al., 2012; Kaushal et al., 2013; Sita et al., 2017; Patriyawaty et al., 2018).

### 3.3 Biochemical adaptation mechanisms involved in mungbean against heat stress

Plants have evolved with a number of biochemical adaptation mechanisms that allow them to tolerate high temperature stress, for example, secondary metabolites, ROS scavenging system and heat-shock proteins (HSPs) (Nakamoto and Hiyama, 1999; Sakamoto and Murata, 2002; Wahid et al., 2007; Mittler et al., 2012). Plants use both non-enzymatic and enzymatic ROS scavenging defense mechanisms to combat ROS production. Non-enzymatic antioxidants such as glutathione (GSH) and ascorbic acid (ASC) collaborate with enzymatic antioxidants such as ascorbate peroxidase (APX), Catalase (CAT), superoxide dismutase (SOD), peroxidase (POX), and glutathione reductase (GR) to maintain high antioxidant levels required for plant heat tolerance. (Suzuki et al., 2012; Awasthi et al., 2015). Isoprenoids, flavonoids, and carotenoids are secondary metabolites that inhibit peroxidase activity and promote high-temperature stress tolerance (Havaux, 1998; Loreto et al., 1998; Rivero et al., 2004). Chickpea grown under high-temperature stress at 35/25 and 45/35°C (day/night, 12 h/12 h) conditions exhibited increased levels of antioxidants such as proline and glutathione (Kumar et al., 2011). The non-enzymatic and enzymatic antioxidant pools are the most efficient and prominent defense mechanisms used by plants. Antioxidants with low molecular weight decrease oxidants without causing significant pro-oxidant activity.

The enormous generation of ROS occurs during heat stress which destroy proteins, lipids, nucleic acids, and carbohydrates (Bita and Gerats, 2013). Mungbean plants can protect themselves from ROS by activating various types of non-enzymatic and enzymatic defensive mechanisms in different cells in plants (Bita and Gerats, 2013). In an experiment under field conditions for heat stress, out of forty-one mungbean genotypes, only a few were found to be heat-tolerant viz., EC693357, ML1299, EC693369, Harsha, and EC693358 which suffered less oxidative damage than heat sensitive mungbean genotypes (Sharma et al., 2016) and when compared to susceptible genotypes, there was increased APX activity in heat resistant genotypes. However, the activity of CAT was increased in both heat-resistant and heat-tolerant genotypes (Sharma et al., 2016). As a result, mungbean genotypes that can withstand under heat stress during reproductive stages could be chosen for screening growth and productivity analysis. Some supplements, such as Ca, K, Mg, and N have been shown to reduce the harmfulness of ROS in plant cells by increasing anti-oxidants such as POD, SOD and CAT (Waraich et al., 2012). Mungbean genotypes can be chosen based on the quantity of enzyme expression, with stress tolerant genotypes having more prominent activities than sensitive genotypes for breeding stress tolerant mungbean cultivars (Kumar et al., 2013).

Aside from the anti-oxidative system, accumulation of various appropriate protective osmolytes such as sugar and their derivatives (polyols), ammonium-based products, proline, and some compounds of sulphonium-based derivatives have been shown in the protection and/or repair of molecules and structures damaged by ROS, as well as in ROS sequestration (Sairam and Tyagi, 2004; Miller et al., 2007). As a result, osmotic adjustment is considered as one of the most promising mechanisms for drought and heat which can be accomplished by accumulating appropriate solutes (e.g., glycine betaine and proline) in protoplasm (Chaves et al., 2003; Bartels and Sunkar, 2005). Proline is one of the prominent osmo-protectant, which also play a crucial role in cellular homeostasis. It also acts as a signaling molecule in triggering specific gene expression, in cell proliferation or cell death, and to alter mitochondrial functions. These are crucial processes for plants’ recovery after stress. Certain natural combinations of osmolytes are formed under abiotic stress conditions such as salt, drought, and heat (Hare et al., 1998; Sakamoto and Murata, 2002). Plants may protect themselves by the accumulation of these solutes in order to increase the tolerance up to certain circumstances. These osmolytes help in increasing the stability of membrane bilayer and proteins. Defensive molecules such as flavonoids, anthocyanin, and plant steroids have been recognized as secondary metabolites that contribute to heat stress tolerance in plants. In response to heat stress, plants also increase their levels of several major phytohormones including ethylene (ET), salicylic acid (SA), and abscisic acid (ABA) to enhance their tolerance. Evaluation of these plant hormones in response to heat stress involves measuring various biochemical and physiological parameters, as well as assessing growth regulating parameters such as photosynthesis and biomass. Additionally, other hormones like auxin, gibberellins (GAs), cytokinins, abscisic acid, brassinosteroids (BRs), and jasmonic acid (JA) have been identified as contributing to heat stress tolerance in plants, as noted by various studies (Peleg and Blumwald, 2011; 2011; Mittler et al., 2012; Zhou et al., 2014; Dobrá et al., 2015; Xia et al., 2015).

### 3.4 Production of heat shock and regulatory protein in response to heat stress

Plants possess an adaptive mechanism to manage heat stress via the production of stress proteins like heat shock proteins (HSPs). These HSPs act as molecular chaperones and assist in activities like protein folding, translocation, aggregation, and degradation in both regular and stressful environments (Vierling, 1991). When subjected to sudden or constant temperature variations, plants increase their HSP production (Schoffl et al., 1998; Nakamoto and Hiyama, 1999), highlighting the critical nature of HSPs when it comes to temperature stress across all species (Vierling, 1991). Plants in dry and semi-arid regions can synthesize and store HSPs in large quantities. HSPs are only present during specific developmental stages like pollen formation, germination, fruit maturation, and embryogenesis. These proteins protect other proteins from denaturation that could be caused by high temperatures. In sensitive organs and tissues where HSPs accumulate quickly, they can safeguard the metabolic system of the cells, which makes them crucial in a plant’s stress response and overall survival. Numerous research studies have established a close relationship between the development of heat tolerance and the synthesis and buildup of HSPs. An instance of this is the elevated expression of HSP68, which is typically expressed constitutively in mitochondria, when potato, maize, tomato, soybean, and barley cells were placed under heat stress (Neumann et al., 1993). HSP70, extensively studied, is believed to partake in several functions, including but not limited to the folding or assisting of proteins, translocation, protein translation, proteolysis, inhibition of aggregation, as well as the restoration of denatured proteins (Zhang et al., 2005). Many plant species necessitate the induction of HSP70 for heat tolerance of cells and tissues post heat stress. Additionally, HSP101 has been deemed vital (Schoffl et al., 1998). Furthermore, different tissues within the same plant manifest diverse abilities to produce specific proteins at 40°C and the magnitude and duration of the synthesis also differ. Five different types of heat-stress protein/chaperone families are reported; these are HSP100, HSP90, HSP70, HSP60 and the small heat-stress proteins (sHSPs) family (Wang et al., 2004), that play an important role in heat-stress mitigation, including protecting native proteins against denaturation.

### 3.5 Mitigating heat stress through agricultural practices

Along with development of tolerant mungbean genotypes integration of heat stress mitigation strategies through appropriate agricultural practices will have greater impact on sustaining the mungbean production amid rising global temperature. Agricultural practices such as timely sowing of crop, selection of early short duration genotypes to escape peak heat stress period, seed priming to enhance the seed vigour and initiate heat tolerance defense mechanisms, irrigation management to enhance water use efficiency and maintain plant water potential, mulching and enhancing organic material to enhance soil water retention and reduce soil temperature. Mungbean sown during summer/spring season, particularly in northern part of India, face more threat of heat stress during later stage of crop growth (HanumanthaRao et al., 2016). To overcome the terminal heat stress, early maturing mungbean cultivars with early seedling vigour are very crucial for sustaining mungbean production. Majority of mungbean cultivars matures in 60–70 days, however, the evaluation of mungbean collections reveals availability of mungbean germplasm which matures within 50 days of sowing (Gayacharan et al., 2020). Seed priming is another promising tool for induction of artificial stress memory response in terms of accumulation of secondary metabolites, antioxidants, improved water plant water potential, etc. (Ahmad et al., 2021; Chakraborty and Dwivedi, 2021; Tamindžić et al., 2023). Utilization of heat tolerant mungbean cultivars may further help in mitigating impact of heat stress in mungbean cultivation. Mungbean cultivars such as Pusa Vishal, SML 668, IPM 99-125, and few more listed in Table 1 are helping in sustaining mungbean cultivation during the summer/spring season in northern parts of India.

## 4 Signaling pathways/factors involved in heat stress tolerance in plants

Plants can detect even a slight deviation in temperature because of the sensing mechanism present on cell membrane through change in fluidity of membrane bilayer. This leads to the conformational changes and post-translation modifications such as phosphorylation/dephosphorylation processes (Kaushal et al., 2016; Sehgal et al., 2016). In response to heat stress plants activate various signaling pathways that lead to the induction of heat stress tolerance genes. There are four major prominent signaling pathways viz. ABA, MAPK, ROS and Ca^2+^. Under high temperatures, ABA levels increase in plant tissues, leading to the activation of stress specific ABA signaling pathway. The ABA signaling pathway operates through ABA binding to receptors, which subsequently engage phosphatases and kinases to regulate downstream gene expression. In plants, this pathway triggers the activation of heat stress tolerance genes, such as HSP70, HSP90, and HSP101 (Batcho et al., 2020; Priya et al., 2020). Similarly, the MAPK signaling cascade plays a critical role in activating MAPKs, which then phosphorylate transcription factors and trigger the induction of stress-responsive genes such as HSP17, HSP26, and HSP70 under heat stress in mung beans (Wang et al., 2021). Additionally, the ROS signaling pathway has been linked to HSP70 and HSP90 induction under heat stress conditions, and it also interacts with other pathways like ABA and MAPK to regulate gene expression (Banti et al., 2010).

Calcium (Ca^2+^) is a ubiquitous secondary messenger that plays a critical role in plant stress responses, including heat stress. Under high temperatures, the heat shock reaction (HSR) is initially triggered by the detection of temperature increase by plasma membrane which then activates ion channels like Ca^2+^ channels and induces an inward flux of Ca^2+^ into cells (Bokszczanin et al., 2013). According to several mechanisms reported, blockage of calcium channel or chelators causes inward flux of Ca^2+^ ions which is a significant signal of heat stress. Ca^2+^ interact with downstream targets, such as calmodulin and calcium-dependent protein kinases (CDPKs), leading to the activation of heat shock transcription factors (HSFs) and several other transcriptional factors such as WRKY39 and stress-responsive genes (Li et al., 2011; 2010). In addition, this Ca^2+^ influx induces the activation of another signaling cascade system including calcium-dependent protein kinases (CDPKs), mitogen-activated protein kinases (MAPKs), and NADPH oxidase, all of which cause the generation of ROS (Sangwan et al., 2002; Suzuki et al., 2011) (Figure 4). These different signaling and defensive pathways are evolutionary conserved processes in legumes and work similarly in almost all of the legume crops. In plants, the Ca^2+^ signaling pathway has been shown to be involved in the induction of heat stress tolerance genes, such as HSP70 and HSP90 (Zheng et al., 2020).

Heat stress activates various other signaling molecules such as PIPK (phosphatidylinositol-4-phosphate-5-kinase), PLD (phospholipase-D), phosphatidic acid, IP3 (D-myo-inositol-1,4,5-triphosphate), and PIP2 (phosphatidylinositol-4,5-bisphosphate). In plants, the activation of two UPR (Unfolded protein response) signaling pathways is triggered by heat stress, one in ER and the other in cytoplasm (Sugio et al., 2009; Pincus et al., 2010; Deng et al., 2011). This leads to proteolytic cleavage in the membrane of the endoplasmic reticulum and activates different bZIP transcription factors (Tfr). (Che et al., 2010; Deng et al., 2011). The accumulation of chaperones in ER, along with calcium signaling, activates brassinosteroid signaling, which in turn activates the transcription of heat-tolerant genes (Che et al., 2010). In cytosol, the unfolded proteins activate the cytosolic UPR pathway for HSF and HSFA2 transcription factors to induce downstream heat stress responsive genes (Sugio et al., 2009).

Plants also use phytohormones, such as ABA and brassinosteroids, as well as signaling molecules like nitric oxide to help them tolerate heat stress (Hasanuzzaman et al., 2011; Asthir, 2015). Studies have shown that applying exogenous ABA to *Phragmites communis* led to a decrease in MDA and H_2_O_2_ content and an increase in POX, APX, CAT, and SOD levels, which suggests less oxidative damage in treated plants compared to non-treated plants (Ding et al., 2010). Similarly, spraying BRs on *Phaseolus vulgaris* increased yield and quality of pods, total phenolic acids in pods, and vegetative growth through the BRs signaling pathway (El-Bassiony et al., 2012). Salicylic acid (SA) is also an important signaling molecule that influences plant growth and development under stress conditions and can act as a protectant under heat stress (Yuan et al., 2008; Hasanuzzaman et al., 2013). Applying SA can increase enzyme activity, carotenoid and chlorophyll levels, photosynthetic rates, ion uptake, flower induction, plant growth, and thermogenesis, and affect the ethylene biosynthesis pathway (Bajguz and Hayat, 2009).

Under heat stress tolerance, nitric oxide (NO) is considered as an another important signaling molecule that regulates various morpho-physiological and biochemical processes in a systematic concentration-dependent manner and acts as a redox-related signaling molecule in legumes (Hasanuzzaman et al., 2013; 2012; 2011; Waraich et al., 2012; Fancy et al., 2017). Exogenous treatment of NO on heat-stressed plants increases the stability and shelf life of chlorophyll molecules, decreases H_2_O_2_ content, and increases antioxidant enzyme activities by enhancing sodium nitropruside levels in plants (Yang et al., 2006).

## 5 Molecular markers and candidate genes associated with heat stress tolerance in plants

The traditional breeding programs are crucial for the identification of stable genetic and genomic resources for their introduction into elite cultivars in addition to advances in biotechnological and molecular approaches (Cabello et al., 2014). Recent efforts on developing new breeding methods have not been enough to cultivate heat tolerant varieties (Grover et al., 2013). Therefore, development of heat tolerance variety is limited through breeding approach. In order to fill this gap, a unique approach has been adapted by breeders for cultivation of stress tolerance variety which is beneficial for developing stress tolerant genotypes with high productivity by interpreting the genomic regions on chromosomes responsible for tolerance (Driedonks et al., 2016). Mungbean is one of the legumes which has rarely received the application of new breeding tools (NBTs) and technologies for understanding the basic tolerance mechanism. However, recent advancement in genome sequencing technologies and their implication in crop research programs has also helped in generating genomic resources in mungbean. Currently there are two genome assemblies (Vradiata_ver6, ASM158444v1) with full genome representation and one assembly (ASM18089v1) with partial genome representation (Tangphatsornruang et al., 2009; Kang et al., 2014; Liu et al., 2016). The genome sequence information is helping in understanding the underlying molecular mechanisms involved in trait expressions.

The utilization of genome sequencing data and advanced genome annotation tools has facilitated the identification of key candidate genes that play essential roles in heat stress tolerance. The genes HSP60, HSP70, HSP90, HSP100, and smHSP are well-known molecular chaperones that are induced by heat stress and are critical in safeguarding plants against heat damage (Kotak et al., 2007; Mishra et al., 2018). Additionally, CBF/DREB1 protein family members and LEA proteins have also been identified as stress-responsive proteins that protect plants from abiotic stresses, including heat stress (Li et al., 2020). Another group of stress-responsive protein that is involved in protecting plants from heat stresses is LEA (Late embryogenesis abundant). In mungbean, some LEA genes viz. *VrLEA-55, VrLEA-47, VrLEA-40,* and *VrLEA-2* were identified by Singh et al. (2022), and reported their upregulation under heat stress conditions. Furthermore, genes encoding antioxidant enzymes such as APX, CAT, and SOD have been identified as significant candidates that aid in scavenging reactive oxygen species under stress conditions. A comprehensive list of identified candidate genes responsible for heat stress tolerance in various plant species is presented in Table 2.

Plant breeding has become more efficient with the aid of Marker aided selection (MAS), however, simple sequence repeats (SSRs) and single nucleotide polymorphisms (SNPs) are commonly used for quantitative trait loci (QTL) analysis in breeding programs (Das and Rao, 2015). Recent advances report that novel breeding approaches utilizing QTL mapping have been effective in developing heat stress tolerance in plants (Jha et al., 2014; Shamsudin et al., 2016). Several QTLs for various traits associated with heat stress tolerance have been identified in different crops. For example, in cowpea, foundation genomic areas linked to heat stress tolerance were identified using SNP markers in a RIL population (CB27 9 IT82E-18), while QTLs associated with browning of seed coats were also discovered (Lucas et al., 2013; Pottorff et al., 2014). In lentil, two QTLs (*qHt-ps* and *qHt-ss*) were characterized, which were linked to heat stress tolerance in pod set and seedling survival (Singh et al., 2016). Similarly, in chickpeas, eight QTLs were identified, four of which were located on the CaLG05 genomic region, for pod set, pod filling, seed number, and grain yield with a combined phenotypic variation of up to 50% under heat stress tolerance, while as the remaining QTLs were located on the CaLG06 genomic regions. (Paul et al., 2018). Jha et al. (2021) identified 37 major and 40 minor QTLs for heat stress tolerance using an inbred population in chickpea. They also identified 32 potential candidate genes in the QTL regions that encode HSPs and HSFs and are involved in the regulation of flowering time and pollen development.

Similarly, in non-legume crops such as wheat plants, QTLs associated with grain filling and leaf senescence were reported on chromosome number 5A and 1B (Yang et al., 2002; Mason et al., 2010). Nine QTLs for tillering and grain filling, and three QTLs for green color were also identified in wheat (Kumar and Wigge, 2010). In maize, six QTLs for pollen tube growth and five QTLs for pollen germination, as well as six QTLs for cellular membrane stability, were detected using RFLP mapping technique (Ottaviano et al., 1991; Frova and Sari-Gorla, 1994). These QTLs and genes are evolutionarily conserved and exhibit mostly similar function in all legumes and other crops. A brief summary of different QTLs/genes associated with heat stress tolerance in legumes/crops is mentioned in Table 2. Currently, the identification and characterization of marker genes associated with heat stress tolerance studies have gained more interest and require better attention and understanding in this field.

## 6 Conclusion and future perspective

Mungbean is a short-duration crop that thrives in a variety of soils and climates. However, its cultivation is increasingly being influenced by heat stress all over the world. To overcome drastic rise in global temperature amid climate change, a comprehensive and holistic approach is required to sustain crop production. The existing variability in *ex situ* collections conserved in seed genebanks or *in situ* on-farm should be the first target to find sources of heat tolerance. Further, utilization of such germplasm to incorporate various climate-smart features into mungbean through new breeding tools would enable mungbean cultivars to perform well in a variety of locations and adapt to diverse agro-climatic regions. In addition to recent advances in new breeding tools, the latest developments in genomics, transcriptomics and metabolomics fields could make a great impact on trait identification and may help to accelerate in developing desired mungbean cultivars. Regions of the genome linked to advantageous characteristics, heat tolerance, water-use efficiency, and the photosynthetic pathways, can also be targeted by combining the genome sequence and phenotyping data. Additionally, a thorough examination of the mungbean pan-genome variability should be performed to understand pan-genome variability at species level. Researchers are now well equipped to explore and exploit underlying molecular processes, which may pave the way for developing multi-stress tolerant mungbean that is best suited to adverse growing environments. Modern scientific tools such as mutational breeding and genome editing may prove very useful in creation of desired novel variability and development of heat tolerant genotypes. Integration of suitable agricultural practices to mitigate heat stress may further provide additional protection to crop cultivation amid rising global temperature.
